# Advancements in Antimicrobial Surface Coatings Using Metal/Metaloxide Nanoparticles, Antibiotics, and Phytochemicals

**DOI:** 10.3390/nano15131023

**Published:** 2025-07-01

**Authors:** Preetha Ebenezer, S. P. S. N. Buddhika Sampath Kumara, S. W. M. A. Ishantha Senevirathne, Laura J. Bray, Phurpa Wangchuk, Asha Mathew, Prasad K. D. V. Yarlagadda

**Affiliations:** 1 School of Mechanical, Medical and Process Engineering, Faculty of Engineering, Queensland University of Technology, Brisbane, QLD 4000, Australia; preetha.ebenezer@hdr.qut.edu.au (P.E.); buddhika.naidelage@hdr.qut.edu.au (S.P.S.N.B.S.K.); s2.senevirathne@qut.edu.au (S.W.M.A.I.S.); laura.bray@qut.edu.au (L.J.B.); 2 Centre for Biomedical Technologies, Queensland University of Technology, Brisbane, QLD 4000, Australia; 3 Australian Research Council Training Centre for Multiscale 3D Imaging, Modelling, and Manufacturing, Queensland University of Technology, Brisbane, QLD 4000, Australia; 4 College of Science and Engineering, James Cook University, Smithfield, QLD 4878, Australia; phurpa.wangchuk@jcu.edu.au; 5 School of Engineering, University of Southern Queensland, Springfield, QLD 4300, Australia

**Keywords:** antibacterial resistance, bacterial infection, biomaterials, biocompatibility, coatings, metal ions, nanoparticles, phytochemical compounds, sustainable

## Abstract

The growing prevalence of bacterial infections and the alarming rise of antimicrobial resistance (AMR) have driven the need for innovative antimicrobial coatings for medical implants and biomaterials. However, implant surface properties, such as roughness, chemistry, and reactivity, critically influence biological interactions and must be engineered to ensure biocompatibility, corrosion resistance, and sustained antibacterial activity. This review evaluates three principal categories of antimicrobial agents utilized in surface functionalization: metal/metaloxide nanoparticles, antibiotics, and phytochemical compounds. Metal/metaloxide-based coatings, especially those incorporating silver (Ag), zinc oxide (ZnO), and copper oxide (CuO), offer broad-spectrum antimicrobial efficacy through mechanisms such as reactive oxygen species (ROS) generation and bacterial membrane disruption, with a reduced risk of resistance development. Antibiotic-based coatings enable localized drug delivery but often face limitations related to burst release, cytotoxicity, and diminishing effectiveness against multidrug-resistant (MDR) strains. In contrast, phytochemical-derived coatings—using bioactive plant compounds such as curcumin, eugenol, and quercetin—present a promising, biocompatible, and sustainable alternative. These agents not only exhibit antimicrobial properties but also provide anti-inflammatory, antioxidant, and osteogenic benefits, making them multifunctional tools for implant surface modification. The integration of these antimicrobial strategies aims to reduce bacterial adhesion, inhibit biofilm formation, and enhance tissue regeneration. By leveraging the synergistic effects of metal/metaloxide nanoparticles, antibiotics, and phytochemicals, next-generation implant coatings hold the potential to significantly improve infection control and clinical outcomes in implant-based therapies.

## 1. Introduction

The escalating prevalence of bacterial infections, coupled with the alarming rise of antimicrobial resistance (AMR), has intensified the demand for innovative strategies to prevent and manage infections associated with medical implants and biomaterials. Implant-associated infections (IAIs) pose significant clinical challenges, often leading to implant failure, prolonged hospitalization, and increased healthcare costs [1]. The adhesion and colonization of bacteria on metallic implant surfaces represent a critical concern in the context of biomaterial-associated infections, particularly in load-bearing medical devices. In hospital environments, opportunistic pathogens, including *Staphylococcus aureus* (*S. aureus*), *Staphylococcus epidermidis*, *Streptococcus sanguinis* (*S. sanguinis*), *Pseudomonas aeruginosa* (*P. aeruginosa*), *Escherichia coli* (*E. coli*), and species of *Porphyromonas*, exhibit a high affinity for biomaterial surfaces, frequently contributing to implant-related infections [2,3]. Traditional systemic antibiotic therapies are frequently inadequate due to the protective nature of biofilms and the emergence of multidrug-resistant (MDR) bacterial strains. Consequently, the development of antimicrobial coatings for medical implants has emerged as a promising approach to mitigate these complications by providing localized, sustained antimicrobial activity at the implant–tissue interface.

Surface modification of implants has consequently gained attention as a promising strategy to enhance antimicrobial performance and mitigate infection risks. Multidisciplinary research efforts have played a pivotal role in advancing surface engineering techniques aimed at improving implant integration while minimizing microbial adherence [4]. Despite the implementation of established infection-control protocols and perioperative antibiotic prophylaxis, such measures have proven insufficient in fully eliminating biomaterial-associated infections (BAIs) [1]. Traditional systemic antibiotic therapies are frequently inadequate due to the protective nature of biofilms and the emergence of multidrug-resistant (MDR) bacterial strains. Consequently, the development of antimicrobial coatings for medical implants has emerged as a promising approach to mitigate these complications by providing localized, sustained antimicrobial activity at the implant–tissue interface.

Over the past two decades, numerous innovative approaches have been explored to modify implant surfaces, with key objectives including the inhibition of initial bacterial attachment, the eradication of adherent microbes, and the suppression of bacterial proliferation in the peri-implant environment [5]. Collectively, these strategies aim to disrupt the early stages of biofilm formation and reduce the risk of chronic infection. Continued progress in surface functionalization holds significant potential to improve patient outcomes by reducing the incidence of implant-related infections and enhancing the overall safety and efficacy of medical devices in clinical practice [1,4]. These developments underscore the pressing need for alternative and more effective antimicrobial strategies to ensure long-term implant success [5].

Among the various antimicrobial agents explored for surface functionalization, three principal categories have garnered significant attention: metal/metaloxide nanoparticles, antibiotics, and phytochemical compounds. Metal/metaloxide nanoparticles, such as silver (Ag), zinc oxide (ZnO), and copper oxide (CuO) CuO, exhibit broad-spectrum antimicrobial activity through multiple mechanisms, including the generation of reactive oxygen species (ROS), disruption of bacterial cell membranes, and interference with intracellular components [4]. These multifaceted modes of action reduce the likelihood of resistance development and have been effectively incorporated into implant coatings to prevent bacterial colonization and biofilm formation [5]. Antibiotic-based coatings offer the advantage of targeted, localized drug delivery, thereby minimizing systemic side effects and achieving high local concentrations at the implant site. However, challenges such as burst release kinetics, potential cytotoxicity, and the diminishing efficacy against MDR strains limit their long-term effectiveness. Moreover, the overuse of antibiotics in implant coatings may contribute to the further development of resistance, underscoring the need for alternative or adjunctive strategies. Phytochemical compounds, derived from plant sources, represent a sustainable and biocompatible alternative for antimicrobial coatings. Compounds such as curcumin, eugenol, and quercetin have demonstrated antimicrobial, anti-inflammatory, and antioxidant properties. Their ability to modulate multiple bacterial targets simultaneously reduces the potential for resistance development. Additionally, phytochemicals can promote osteogenic differentiation and tissue regeneration, offering multifunctional benefits for implant integration.

The integration of these antimicrobial agents into implant coatings necessitates a multidisciplinary approach, combining insights from materials science, microbiology, and clinical medicine. Advancements in nanotechnology and surface engineering have facilitated the development of coatings that not only prevent bacterial adhesion and biofilm formation but also support tissue healing and implant longevity. Future research should focus on optimizing the synergistic effects of combined antimicrobial agents, assessing long-term biocompatibility, and evaluating clinical outcomes to establish effective and durable solutions for implant-associated infections.

We conducted a structured narrative review guided by recommendations for narrative literature synthesis. Key search terms (“antimicrobial coatings”, “metal/metaloxide nanoparticles”, “phytochemicals”, “implant surface functionalization”, “biofilm”) were used across PubMed, Scopus from 2000 until Jan 2025. Google Scholar was used as a supplementary resource. Titles and abstracts were screened for relevance; full texts were retrieved via Mandeley reference library to build a thematic evidence base. Studies were included if they discussed surface roughness, chemistry, and antibacterial agents on implants; non-English articles, reviews, and conference abstracts were excluded. We organized findings into three themes: metal/metaloxide nanoparticles, antibiotic, and phytochemical coatings. Within each theme, we summarized mechanisms of action, biocompatibility, and limitations. A summary matrix captured study design, materials, outcomes, and notable gaps. Critical appraisal focused on methodological rigor and potential bias, drawing from systematic principles where feasible. Limitations of the narrative approach and search boundaries are discussed in the Conclusion.

## 2. Evolution of Surface Modification on Biomaterials

**Figure 1:**
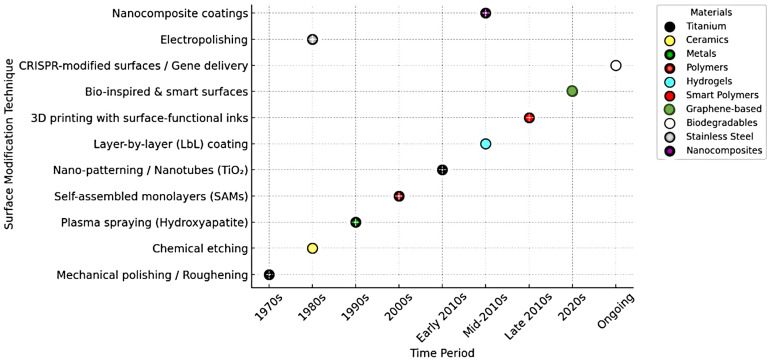
Outlining the evolution of surface modification on biomaterials (1970, 2010 are titanium, 2020s is graphene based).

Initially, the 1970s and 1980s research focused on foundational physical methods such as mechanical polishing, roughening, and chemical etching, primarily applied to metals and the field has progressed significantly. In the 1980s, chemical etching on ceramics and electropolishing was performed on stainless steel gained attention. Moving into the 1990s, plasma spraying with hydroxyapatite gained traction, enhancing osseointegration on titanium surfaces, followed by self-assembled monolayers (SAMs) in the early 2000s, offering controlled surface chemistry often used with metals and ceramics [1,3]. In the early 2010s, nano-patterning and nanotubes (particularly TiO_2_-based) began emerging, leveraging titanium and graphene-based materials for cellular-level surface interactions [4]. This was succeeded in the mid-2010s by layer-by-layer (LbL) coatings, widely used with hydrogels and smart polymers for customizable, multi-functional surfaces. From the late 2010s onward, the field saw the introduction of 3D printing with surface-functional inks, enhancing spatial control and material diversity, particularly within biopolymers and nanocomposites [5]. More recently, bio-inspired and smart surfaces have emerged, aiming to mimic natural tissue environments and adapt dynamically to physiological changes [6]. Currently, the frontier of surface modification lies in CRISPR-modified surfaces and gene delivery systems, signaling a shift toward biodegradable materials and gene-level modulation for personalized and regenerative therapies. This progression underscores a broader trend in biomaterials science: from passive surface modification toward active, bio-responsive, and genetically tune able systems aimed at enhancing clinical performance and integration [6,7,8]. Figure 1 illustrates the chronological development of surface modification techniques in biomedical materials, spanning from the 1970s to the present. Each technique is plotted against the time-period of its emergence or predominant use, along with the material type it is most associated with. The timeline reveals a clear evolution from basic physical methods to sophisticated, bio-functional, and gene-driven strategies. Each marker not only signifies a technique and its era but also highlights the material’s growing complexity and functional integration over time.

When an implant is introduced into the body, the initial stage of healing is initiated by coating the implant surface with a protein layer facilitated by the host body mechanisms and body fluid [6]. This protein adsorption creates a conducive environment for microbial attachment and colonization on the implant surface. Consequently, the physicochemical properties of the substrate significantly influence microbial colonization [7]. Research has demonstrated that biomaterial exhibits higher protein absorption, thereby increasing the likelihood of bacterial colonization [8]. A promising approach to prevent implant-associated infections involves the prevention of bacterial adhesion [9].

The evolution of surface modification techniques aimed at combating infections, particularly on metal surfaces, is a subject of profound interest and significance within biomedical research [10]. Preventing bacterial adhesion is crucial, as it serves as the first step in biofilm formation and acts as a primary defence mechanism for bacterial firm colonization [10,11]. The physicochemical properties of biomaterials, including roughness, hydrophilicity, hydrophobicity, and surface charge, play a significant role in influencing bacterial adhesion and biofilm formation [12]. These modifications are tailored to suit diverse applications, depending on the specific type of biomaterial employed. In the early stages, surface modification research focused on understanding the impact of surface micro-roughness in reducing bacterial adhesion and colonization on metallic surfaces [13]. Studies revealed that most Gram-negative bacterial species exhibit a super-repulsive nature, while Gram-positive bacterial species tend to adhere to these surfaces [14].

Furthermore, comparative studies for adhesion characteristics of Gram-positive bacteria, *S. aureus*, and Gram-negative bacteria, *P. aeruginosa*, on metallic surfaces with varying roughness revealed significant differences in adhesion or colonization [15]. Studies indicated that metallic surfaces with a roughness of less than 30 nm and exceeding 2 µm could enhance the adherence of several strains of clinical pathogens, subsequently contributing to biofilm synthesis [16]. Furthermore, research findings demonstrated that bacterial growth reduction of nearly 35% was observed among *Staphylococcus* variants on metallic surfaces after physical characteristics modifications [17]. It was observed that 98% of *S. aureus* could colonize and adhere to ultra-smooth surfaces even with roughness below 0.5 nm, whereas *P. aeruginosa* struggled to colonize surfaces with roughness below 1 nm. Unfortunately, the intricate mechanism of microbial repulsion on super-hydrophobic surfaces remains incompletely understood [18].

The continuous development of surface modification techniques offers promising advancements in medical implant technologies. By refining biomaterial surfaces to inhibit bacterial colonization while maintaining biocompatibility, researchers can significantly enhance the safety and efficacy of medical devices, ultimately leading to improved patient outcomes [19]. To further enhance defence mechanisms against bacterial colonization, any bacteria reaching the surface could be promptly eliminated through additional coating on surfaces. Studies have demonstrated the effectiveness of these advanced surface modifications in reducing bacterial adherence and improving the antimicrobial properties of biomaterials.

## 3. Coatings as Surface Modification

Among the array of surface modification approaches, antibacterial agent coatings have emerged as a prominent solution owing to their inherent anti-adhesive properties. Coating generally is creating an additional layer over the surface of the material without disrupting the natural property of the material [20]. By creating a barrier that inhibits bacterial adhesion, these coatings effectively reduce the likelihood of biofilm formation, a primary precursor to infection. Numerous strategies for surface modification aimed at generating coatings with anti-infection properties have been documented [21]. These approaches share a common principle, aiming to prevent bacterial attachment to the surface, eliminate bacteria in direct contact with the surface, and impede bacterial growth away from the implant surface [22]. This approach leverages the biological properties of the coatings while preserving the mechanical properties of the substrate materials. Hence, the coating should be biocompatible, non-cytotoxic, and help tissue generation promoting tissue integration. Current strategies for anti-infection modifications predominantly include polymeric coatings with anti-adhesive properties, controlled-release depots for antimicrobial agents, and coatings designed for contact-killing effects [23].

Coatings engineered for contact-killing effects represent a promising frontier in the quest for infection-resistant surfaces [3]. Applying a polymer coating is an effective and cost-efficient method to achieve these desirable surface properties. By incorporating bactericidal compounds or integrating antimicrobial agents directly into the coating matrix, they actively eradicate bacteria upon contact, providing an additional layer of protection against infection [20]. By releasing antimicrobial agents in a controlled manner, these depots can target and eliminate bacteria near the implant, further bolstering the defences against infection [24]. Moreover, controlled-release depots for antimicrobial agents offer a precise and localized means of delivering therapeutic compounds.

Various techniques have been employed to achieve coatings (Table 1), including electrophoretic deposition, layer-by-layer deposition, sol-gel methods, casting, dip coating, spin coating, and electrospinning [25]. An electrophoretic deposition colloidal technique using the electrophoresis mechanism involves the movement of charged particles suspended in a solution under an electric field. This method facilitates the ordered deposition of these particles onto a substrate, thereby enabling the development of thick and/or thin films, coatings, and free-standing bodies [3]. Employing a layer-by-layer technique for loading antibacterial agents enables controlled drug release and immediate bacteria elimination upon contact. This method was utilized in the deposition process to produce a gelatine film incorporating vancomycin, significantly prolonging the release duration [26]. Similarly, constructing a tube array with embedded antibacterial agents extends the release time, with larger tube diameters further enhancing this effect.

Various methods of incorporation, such as addition during material elaboration (e.g., cement) [39], absorption in porous biomaterials, binding to functionalized coatings, or integration into self-assembling mono/multilayer organic coatings [40], offer diverse approaches to enhance the efficacy of these agents [41]. Disrupting quorum-sensing through natural and synthetic autoinducers may also be employed as a preventive measure against biofilm formation [42]. Depositing antimicrobial peptides is another tactic employed to diminish bacterial adhesion on biomaterial surfaces [43]. Coatings with reduced toxicity can be achieved through the application of bacteriolytic enzymes [44,45].

Non-metallic elements such as iodine and fluorine, as well as inorganic compounds like hydroxyapatite, carbides, nitrates, and various metal ions, can be directly applied as coatings in different concentrations, making them well-suited for a wide range of coating applications [46]. Though inorganic compounds are not as effective as antibiotic coatings, they contribute less to antimicrobial resistance issues. Most inorganic compounds can be coated using several coating methods, such as plasma electrolytic oxidation, plasma ion implantation, electrochemical treatment, sol-gel, or micro-arc oxidation [47]. Thus, the approach of coating the implant surface has become one of the most suitable ways to enhance functional properties in implants. Therefore, the selection of the coating methodology mainly depends on the coating agents and their applications.

## 4. Antibiotics as Antimicrobial Agents for Coating

Bacterial colonization is prevented using coatings loaded with antimicrobial agents, which can be either organic or inorganic elements, antibiotics, antimicrobial peptides, polymers, or combinations of these compounds [48,49]. Incorporating antibiotics into the coating allows for the attainment of elevated antibiotic concentrations in the targeted region [50]. The utilization of both organic and inorganic antimicrobial agents is an extensively investigated option. Additionally, these established antibiotics exhibit minimal side effects and, ideally, are cost-effective [51]. Antibiotics can be integrated throughout the entirety of the biomaterial or applied in the surface coating, resulting in elevated antibiotic concentrations within the targeted area [52]. Antimicrobial agents employed in coating biomaterials should possess broad-spectrum activity, effectively targeting a range of bacterial and fungal microorganisms commonly associated with Biomaterial-Associated Infections (BAIs) [53] (Table 2).

Various antibiotic coating techniques have been developed to combat post-surgical infections associated with biomedical implants. One of the most commonly used methods is dip coating, which involves immersing the implant into a solution containing antibiotics. It is simple, cost-effective, and scalable for mass production, making it a preferred choice for orthopedic and dental implants [68,69]. Similarly, spin coating provides high uniformity and precise thickness control by depositing a thin liquid film on a rotating substrate. This technique is ideal for coating microdevices and bioelectronic implants [70,71].

Electrophoretic deposition (EPD) applies an electric field to deposit charged antibiotic-loaded particles or polymers onto conductive implant surfaces. This technique allows excellent control over film thickness and is compatible with a range of implant materials like titanium and cobalt-chrome [72,73]. Layer-by-layer (LbL) assembly, on the other hand, enables nanometer-scale control by sequentially adsorbing oppositely charged polyelectrolytes. It offers the advantage of customizable and sustained drug release and is commonly applied to cardiovascular stents, catheters, and antimicrobial surfaces [70,74].

The sol-gel technique incorporates antibiotics into bioactive glass or silica matrices, forming thin, bioactive, and antibacterial coatings through low-temperature processing. It is suitable for bone and dental implants due to its ability to support osseointegration and localized drug delivery [75,76]. In contrast, plasma spraying, often used to deposit hydroxyapatite mixed with antibiotics, is a high-temperature process that creates thick, adherent coatings ideal for long-term orthopedic applications. However, it is less suitable for heat-sensitive drugs [77,78].

Finally, supercritical fluid coating uses supercritical CO_2_ as a solvent to load antibiotics into porous implant surfaces. This solvent-free and low-temperature technique preserves the activity of antibiotics and is particularly effective for coating porous titanium or biodegradable polymer implants [79]. These diverse coating strategies provide tailored solutions for preventing implant-related infections while promoting tissue integration and long-term functionality.

The initial success in preventing and locally treating infections was during hip replacement surgeries, which was achieved by incorporating antibiotics into the bone cement. Presently, the common practice in arthroplasty surgery involves the use of non-biodegradable polymethyl-methacrylate (PMMA) bone cement infused with antibiotics [67]. Initially utilized as an anchor for joint replacements, this approach has evolved and gained acceptance as a local drug-release device [80]. Successful loading of erythromycin at varying concentrations onto niobium oxide coatings on stainless steel was achieved through a modified sol-gel route [66]. Consequently, antibiotic-coated catheters with these primary antibiotic coatings emerge as a promising approach to prevent catheter-associated infections [81].

Though the release of antibiotics is not solely determined by surface area and matrix diffusion, it is also influenced by various factors, including pH, pore formation, swelling, and degradation mode [68]. However, a challenge arises as numerous antibiotics are released at a significant rate over a brief duration, potentially causing tissue toxicity. Dependence on high doses of antibiotics is strictly limited due to adverse effects on patients [80]. Therefore, to mitigate the risk of resistance, it is advisable to utilize a combination of antimicrobials [71]. However, the subsequent emergence of antibiotic-resistant bacterial species has led to a resurgence of frequent and challenging infections.

Bacterial resistance to antibiotics remains a formidable challenge in modern medicine, necessitating innovative strategies to counteract this threat. Three pivotal mechanisms have been identified to combat bacterial resistance: inhibition of quorum sensing (QS), disruption of biofilm matrices, and inhibition of efflux pumps [72]. These approaches target the fundamental processes that bacteria employ to resist antimicrobial agents. Quorum sensing is a cell-to-cell communication mechanism that bacteria use to coordinate gene expression, including virulence factors and biofilm formation, based on population density. Disrupting QS pathways can attenuate bacterial pathogenicity without exerting selective pressure that leads to resistance [73]. Recent studies have highlighted the potential of QS inhibitors (QSIs) in mitigating bacterial infections. For instance, Zhao et al. (2020) demonstrated that certain organisms produce analogues of QS signals, which competitively bind to bacterial QS receptors, thereby inhibiting QS-regulated functions [75]. Additionally, Naga and Shaaban (2023) emphasized the role of QSIs in reducing bacterial virulence and biofilm formation, suggesting their utility as alternative therapeutic agents [76].

Biofilms are structured communities of bacteria encased in a self-produced extracellular polymeric substance (EPS) matrix, which confers protection against antibiotics and the host immune system. Targeting the biofilm matrix can enhance the efficacy of antimicrobial treatments [75,76]. Long et al. (2020) reported that elasnin, a compound derived from *Streptomyces mobaraensis*, effectively disrupted the EPS matrix in multispecies biofilms, rendering the bacteria more susceptible to antibiotics [77]. Furthermore, Srivastava et al. (2024) discussed various strategies to inhibit or eradicate biofilms, including the use of natural agents that interfere with EPS components, thereby compromising the structural integrity of biofilms and enhancing antimicrobial penetration [78].

Efflux pumps are transport proteins that bacteria utilize to expel toxic substances, including antibiotics, from their cells, contributing significantly to multidrug resistance. Inhibiting these pumps can restore the intracellular concentration of antibiotics, enhancing their efficacy [77]. Recent research has focused on identifying efflux pump inhibitors (EPIs) that can be co-administered with antibiotics. For example, terpenes such as carvacrol have been shown to inhibit efflux pumps like NorA in *Staphylococcus aureus*, as detailed by a systematic review in 2022. Additionally, strategies involving the modification of antibiotic structures to evade efflux or the use of adjuvants that block pump activity are being explored to combat resistance mechanisms effectively.

Overall, targeting bacterial communication systems, protective biofilm structures, and drug efflux mechanisms offers promising avenues to overcome antibiotic resistance. By disrupting these key processes, it is possible to enhance the susceptibility of bacteria to existing antibiotics and reduce the incidence of resistant infections.

## 5. Metal/Metaloxide Nanoparticles and Ions as Antibacterial Agents for Coating

Metal/metaloxide nanoparticles and their ionic forms are increasingly being used in antibacterial coatings for medical implants due to their unique physicochemical and biological properties. One of the primary advantages is their broad-spectrum antimicrobial activity, including effectiveness against multidrug-resistant (MDR) bacteria. Metal/metaloxide nanoparticles such as silver Ag, Cu, ZnO, and TiO_2_ function through multiple mechanisms: they disrupt microbial cell membranes, generate reactive oxygen species (ROS), interfere with DNA replication, and denature essential proteins [79]. This multifaceted approach greatly reduces the risk of resistance development compared to conventional antibiotics [78].

Another significant benefit is the prolonged antibacterial effect due to the slow and sustained release of metal ions. Silver nanoparticles, for example, can continuously release Ag^+^ ions over time, maintaining a localized antimicrobial environment around the implant without the need for repeated dosing [80]. This extended activity is particularly useful in preventing late-stage infections, which are a common cause of implant failure. In contrast, antibiotic-loaded coatings often suffer from rapid drug depletion, limiting their protective window.

Metal/metaloxide nanoparticles also offer excellent surface modification capabilities. They can be easily incorporated into various coating matrices or directly immobilized on implant surfaces using advanced fabrication techniques such as plasma spraying, electrophoretic deposition, or sol-gel methods. These coatings provide strong adhesion and conformability to different implant materials, enhancing durability and functionality [81]. Additionally, nanoparticle size, shape, and surface charge can be finely tuned to optimize both antibacterial efficacy and host compatibility.

A further advantage is their role in inhibiting biofilm formation, a major contributor to chronic implant-associated infections. Metal/metaloxide nanoparticles prevent bacterial adhesion and disrupt quorum sensing pathways, which are crucial for biofilm development. This not only hinders the initial colonization of implant surfaces but also limits the establishment of persistent microbial communities [82]. Such anti-biofilm properties are especially beneficial in orthopaedic, dental, and cardiovascular implants, where biofilm-associated infections are challenging to eradicate.

Lastly, metal/metaloxide nanoparticles can be used in synergistic combinations with antibiotics or natural antimicrobial agents to enhance overall antibacterial performance. Co-delivery systems incorporating both metal ions and drugs have demonstrated improved bacterial killing, reduced required dosages, and minimized cytotoxicity. These hybrid strategies capitalize on the strengths of both components, providing a versatile platform for infection-resistant implant design.

While metal/metaloxide nanoparticles (NPs) such as silver, copper, zinc oxide, and titanium dioxide have demonstrated excellent antimicrobial efficacy in implant coatings, several biological and technical drawbacks limit their clinical translation. A primary concern is cytotoxicity. At elevated concentrations or due to uncontrolled ion release, these nanoparticles may induce oxidative stress, mitochondrial damage, and apoptosis in host cells such as osteoblasts and fibroblasts, potentially impairing osseointegration [80]. For example, silver nanoparticles, while potent against pathogens, have a narrow therapeutic window between antimicrobial efficacy and toxicity to human tissues.

Another major disadvantage is the potential for chronic inflammation and immune response. Metal/metaloxide nanoparticles are foreign bodies that can be recognized by immune cells, triggering inflammatory cascades. Persistent inflammation may result in tissue necrosis, fibrous encapsulation, and implant rejection [81]. Moreover, repeated or long-term exposure to these nanoparticles, particularly in degradable or porous implant systems, may lead to systemic distribution and accumulation in secondary organs such as the liver, kidneys, and spleen, raising long-term safety concerns [83].

There is also concern about the emergence of bacterial resistance to Metal/metaloxide nanoparticles. Although metal NPs attack bacteria through multiple pathways, sub-lethal concentrations or prolonged exposure can promote adaptive mechanisms in microbes, such as efflux pumps, reduced membrane permeability, and extracellular matrix production that sequesters ions. This resistance development, while slower than with antibiotics, has been observed particularly with silver and copper nanoparticles under in vitro and in vivo conditions [84].

Furthermore, nanoparticle aggregation and instability pose challenges in maintaining consistent antimicrobial performance. Nanoparticles tend to agglomerate in physiological environments, reducing their surface area and, consequently, their antibacterial efficacy. Environmental factors such as pH, temperature, and protein adsorption (protein corona effect) can further alter nanoparticle behaviour, affecting ion release profiles and reducing bioavailability. This instability makes it difficult to predict long-term effectiveness and may lead to inconsistent outcomes in vivo.

Finally, the manufacturing complexity and cost of incorporating Metal/metaloxide nanoparticles into implant coatings remains a barrier to commercialization. Techniques such as chemical vapor deposition, sputtering, or sol-gel processes require stringent control over nanoparticle size, dispersion, and surface adherence. Scaling up these techniques while maintaining uniformity and biocompatibility is challenging [84]. Moreover, regulatory approval for metal nanoparticle-containing medical devices remains stringent due to concerns over environmental toxicity and patient safety, prolonging development timelines and increasing costs [85]. Some of the research published the advantages of using various Metal/metaloxide nanoparticles as coating are given in Table 3.

However, care should be taken in selecting the concentration of these metals as an antibacterial agent due to its cytotoxicity and highly reactive properties. Hence coating technique like metal doping or plasma electrolytic oxidation followed by ion implantation are highly recommended methods for coating metal nanoparticles [84]. Initial fast release of the compound, followed by slow release, should be maintained below 300 ppm in human blood.

Techniques such as chemical vapor deposition, sputtering, or sol-gel processes require stringent control over nanoparticle size, dispersion, and surface adherence. Scaling up these techniques while maintaining uniformity and biocompatibility is challenging [87]. Moreover, regulatory approval for metal nanoparticle-containing medical devices remains stringent due to concerns over environmental toxicity and patient safety, prolonging development timelines and increasing costs [88]. In conclusion, while metal/metaloxide nanoparticles offer compelling antimicrobial properties for implant coatings, their toxicity, immunogenicity, resistance risks, instability, and cost must be critically evaluated. Future research must focus on optimizing formulations to balance efficacy and safety for long-term clinical application.

## 6. Phytochemical Compounds as Antibacterial Agents for Coating

**Figure 2:**
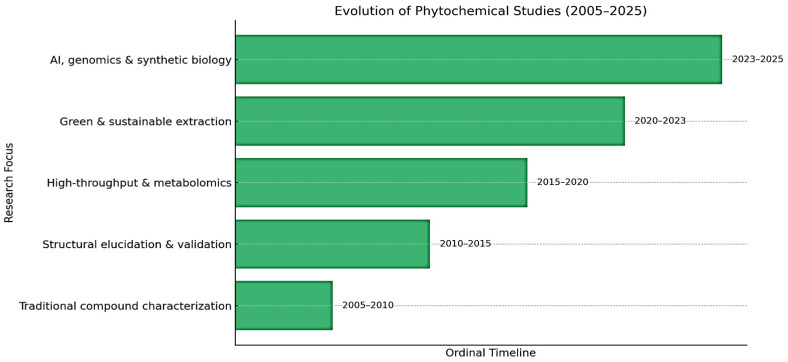
Outlining the chronological progression of research focus in phytochemical studies (2005–2025).

Over the past two decades, phytochemical research has undergone a significant evolution, transitioning from conventional compound identification techniques to advanced integrative approaches. The temporal evolution of methodological approaches in phytochemical research is given Figure 2. Early on, the field was primarily centered on traditional compound characterization, employing classical methods such as chromatography and spectroscopy [94]. This phase was followed by a focus on structural elucidation and validation with increased reliance on nuclear magnetic resonance (NMR), mass spectrometry (MS), and crystallography to confirm the identities of phytochemicals [95]. The emergence of high-throughput technologies and metabolomics enabled large-scale profiling and analysis of phytochemical diversity, thereby accelerating discovery and functional annotation. Subsequently, from 2020 to 2023, the emphasis shifted toward green and sustainable extraction techniques, aligning with environmental priorities and employing methods such as supercritical fluid extraction and green solvents. The most recent phase, spanning 2023 to 2025, reflects the integration of artificial intelligence, genomics, and synthetic biology. These technologies have begun to reshape phytochemical studies by enabling predictive modelling, genome mining for biosynthetic gene clusters, and the engineered biosynthesis of target compounds. This chronological progression illustrates the dynamic nature of phytochemical research, increasingly driven by interdisciplinary innovation and sustainable practices.

Phytochemicals, derived from plant sources, are well-recognized for their inherent antibacterial properties, positioning them as promising candidates for creating coatings that effectively combat microbial colonization [95]. Furthermore, the high biocompatibility of many phytochemicals with mammalian cells ensures a harmonious integration of coated nanowire surfaces with surrounding tissues, thereby reducing the risk of adverse reactions [96]. The anti-inflammatory attributes of certain phytochemicals contribute to minimizing inflammation at the implant site, fostering an environment conducive to optimal healing. Additionally, the osteogenic potential associated with specific plant-derived compounds suggests that coating nanowires with such phytochemicals may augment osseointegration, enhancing the overall effectiveness of implants in orthopaedic and dental applications [97]. The natural antioxidant effects of phytochemicals further contribute to the longevity of implants by protecting tissues from oxidative stress. Importantly, the use of phytochemicals as antibacterial agents presents a potential avenue for reducing the risk of antibiotic resistance, given their complex composition that challenges bacterial resistance mechanisms [98]. Embracing an environmentally friendly approach, the utilization of plant-derived compounds aligns with sustainable practices, leveraging renewable resources for implant coatings. The diverse chemical profiles offered by a plethora of plant-derived phytochemicals empower researchers to tailor coatings to specific antibacterial requirements, addressing challenges posed by different bacterial strains [99].

The ongoing global threat of antibiotic resistance underscores the necessity for less susceptible alternatives to antibiotics [100]. Researchers are employing phytochemicals to urgently establish a robust pipeline of antimicrobials [101]. Drawing from plant-based treatments, numerous phytopharmaceutical enterprises have developed methods for neutralizing or managing resistant bacterial species [102]. These phytochemical molecules deactivate the pathogenicity of microbes by influencing gene expression, regulating virulence, or modifying cell metabolisms [51,103,104,105].

Additionally, the mode of action of plant molecules extends beyond targeting microbes; they can also induce changes in the host immune system response, protect host cells, and expedite the healing process after infections [106]. A study by Wangchuk and his team in 2004 reported that over 73% of modern pharmaceutical drugs are derived from natural green sources and are more effective [107]. According to numerous previous studies, the antimicrobial properties are attributed to the presence of secondary metabolites [108]. These compounds exhibit antibiotic and antimicrobial abilities, interfering with vital components of bacteria, reducing growth, denaturing DNA and RNA, a nd inhibiting protein synthesis. Some compounds attach to the bacterial cell wall, creating pores in the cell wall [109]. Some of the phytochemicals used in bone healing studies are listed in Table 4 below.

## 7. Application of Plant-Based Compounds as Antibacterial Coatings

The escalating antibiotic resistance observed in conventional antimicrobial agents has prompted a shift towards herbal medicine and natural antimicrobial products [121,122]. In 2015, with the introduction of green technology by the UN Sustainable Development Goals, herbal products gained prominence in the research community [36,123]. The resurgence of herbal products symbolizes safety for the environment and humans compared to commercial synthetic compounds. The active substances produced by plants to combat microbial infections or pests are known as phytochemicals [124].

The use of herbal products and their derivatives in primary healthcare has witnessed a significant surge over the past decade. Additionally, these phytochemicals have been observed to influence or modify resistance mechanisms in bacteria [125,126]. Numerous crude plant extracts have been screened for their antibacterial activity [127,128]. Traditional medicines have roots in records of the usage of a combination of one or many plant parts of medicinal plants for treating and preventing infectious diseases [129,130]. Plants produce a broad spectrum of secondary metabolites, including tannins, terpenoids, alkaloids, polyphenols, and flavonoids, all of which have demonstrated antimicrobial properties in vitro against both Gram-positive and Gram-negative bacteria [113,131]. The suggestion is that, alongside these antimicrobials, plants may generate various other chemicals, such as inhibitors of bacterial multidrug resistance pumps, to enhance the permeation of antimicrobials into bacterial cells [107,132].

Presently, both Gram-positive and Gram-negative multidrug-resistant bacteria pose a growing threat in clinical infections [133,134,135,136]. Treating these resistant or tolerant species with antibiotics can be a double-edged sword. For instance, common wild *Staphylococcus* species can mutate into MRSA when exposed to antibiotics for over 11 weeks [113,137]. Therefore, amongst numerous alternative antimicrobials, secondary metabolites derived from plant organisms provide a diverse range of antimicrobial agents [138,139,140,141]. Consequently, herbs with multi-target antibacterial characteristics are often considered the most effective and secure antimicrobial medicines against the multidrug resistance crisis [142,143]. The historical evidence of using whole herbal plant extracts, and parts of herbal plants to treat illnesses, adds value to this thought [144,145].

In contrast to commercial antibiotics, phytochemicals contribute to promoting host-directed therapy, which can combat pathogenic bacterial mutations. They also help avoid drug stress resulting from frequent or high doses of antibiotics [146,147]. Plants synthesize a diverse array of secondary metabolites, encompassing tannins, terpenoids, alkaloids, polyphenols, and flavonoids, which have demonstrated in vitro antimicrobial properties against both Gram-positive and Gram-negative bacteria [148,149,150,151]. Additionally, these phytochemicals have been observed to modulate or alter resistance mechanisms in bacteria [152,153]. Among the wide range of phytochemicals, phenolics, terpenoids, essential oil constituents, alkaloids, lectins, polypeptides, and polyacetylenes are commonly linked to antimicrobial activity [154,155]. It is suggested that, alongside these antimicrobials, plants may produce various other chemicals, such as inhibitors of bacterial multi-drug resistance pumps, that facilitate the permeation of antimicrobials into bacterial cells [76,156]. Moreover, there is a notable lack of a systematic description regarding the structure-property relationship of antibacterial phytochemicals, which could potentially hinder their widespread adoption.

Based on their chemical structures, phytochemicals exhibit various bactericidal mechanisms effective against different types of bacteria [157]. Alkaloids significantly impact the generation of EPS, with piperine, an alkaloid found in peppercorns, aiding MRSA and MSSA in reproducing and forming colonies [158]. Berberine, an alkaloid compound found in the bark, twigs, and rhizomes of Barberry bushes, is known to limit the growth of multidrug-resistant *Staphylococcus aureus* [36]. Plant terpenoids have antibacterial properties, particularly effective against Gram-positive bacteria [154]. They interfere with the respiratory chain, make cells more fluid and permeable, and cause more K+ ions to seep out of cell membranes [144]. Consequently, they contribute to the bacterial cell’s death before EPS is produced. Phenolic substances can effectively damage RNA molecules or halt cells’ RNA transcription processes. The methanolic extracts obtained from spices and herbs such as cumin, fennel seed, anise, ajwan, and ginger have demonstrated effectiveness against both Gram-positive bacteria [155], including *Bacillus amyloliquefaciens* and *S. aureus*, and Gram-negative bacteria like *E. coli* and *P. aeruginosa* [151]. The compound isolated from garlic, allicin, has a broad spectrum against bacteria, including multidrug-resistant enterotoxigenic strains of *E. coli* [36]. Commonly used herbs and spices, including garlic, black cumin, cloves, cinnamon, thyme, bay leaves, mustard, and rosemary, possess essential oils with proven antimicrobial properties [155]. Due to the complex composition of the extracts, containing carbohydrates, inulin, alkaloids, glycosides, flavonoids, terpenoids, tannins, reducing sugars, soluble phenols, and saponin glycosides, attributing the observed antimicrobial activity to a specific constituent is challenging [76,155]. Encouraging results of certain phytochemicals exhibiting bactericidal efficacy against MRSA encourage researchers to use these molecules against other biofilm producers in the urgent biofilm crisis [158].

The escalating antibiotic resistance observed in conventional antimicrobial agents has prompted a shift towards herbal medicine and natural antimicrobial products [36]. Presently, both Gram-positive and Gram-negative multi-drug-resistant bacteria pose a growing threat in clinical infections [155]. Treating these species with antibiotics can be a double-edged sword. For instance, common wild *Staphylococcus* species can mutate into MRSA when exposed to antibiotics for over 11 weeks [159]. Therefore, the demand for alternative strategies to antibiotics and the discovery of novel antibiotics has surged.

Plant-based compounds present a promising and sustainable alternative for antibacterial coatings on biomedical implants. Rich in bioactive phytochemicals such as flavonoids, terpenoids, and alkaloids, these natural agents exhibit broad-spectrum antimicrobial activity while often promoting osteogenesis and tissue regeneration [76,155]. Unlike conventional antibiotics or metal/metaloxide nanoparticles, plant-derived coatings typically offer lower cytotoxicity and reduced risk of inducing bacterial resistance. Their biocompatibility, environmental friendliness, and multifunctional potential make them attractive candidates for next-generation implant coatings. However, to fully realize their clinical potential, future studies must address challenges related to standardization, controlled release, and long-term stability of these bioactive compounds on implant surfaces.

## 8. Comparative Resistance Mechanisms of Bacteria to Antibiotic, Metal/Metaloxide Nanoparticle, and Phytochemical-Based Coatings

Bacterial resistance to antimicrobial agents remains a significant obstacle in implantable biomaterial applications. Conventional antibiotic coatings, while initially effective, are often compromised by well-characterized bacterial resistance mechanisms such as β-lactamase enzyme production, target modification, and active efflux pumps [156]. β-lactamase enzymes hydrolyze β-lactam antibiotics, rendering them inactive, while mutations or post-translational modifications in target molecules such as DNA gyrase (in fluoroquinolone resistance) or penicillin-binding proteins (in methicillin-resistant Staphylococcus aureus) lead to reduced antibiotic binding. Efflux pumps like NorA and AcrAB-TolC actively expel antibiotics from bacterial cells, lowering intracellular drug concentrations and impairing efficacy.

In response to metal/metaloxide nanoparticle (NP) coatings, bacteria also demonstrate adaptive resistance, though the mechanisms differ. Metal/metaloxides NPs such as silver, zinc oxide, and copper exert antimicrobial effects via oxidative stress, membrane disruption, and protein/DNA damage. Bacterial resistance mechanisms include the upregulation of efflux pumps specific to metal ions (e.g., CusCFBA for silver and copper), increased production of extracellular polymeric substances (EPS) to physically block nanoparticle access, and biofilm thickening, which reduces NP penetration. Additionally, reduction of ionic uptake and expression of metal-binding proteins or metallothioneins can detoxify metal ions intracellularly, though such adaptations often come at a high metabolic cost and are slower to evolve compared to antibiotic resistance.

In contrast, phytochemical-based coatings, which utilize plant-derived secondary metabolites such as polyphenols, flavonoids, terpenoids, and alkaloids, exhibit multi-target antimicrobial activity, making the development of resistance substantially more difficult. These compounds can disrupt bacterial membranes, inhibit quorum sensing, interfere with nucleic acid synthesis, chelate essential ions, and inhibit enzymes crucial to metabolism and replication [157]. The multifaceted mode of action leaves fewer escape routes for bacteria to develop resistance, as it would require simultaneous mutations in multiple pathways, which is statistically improbable.

Moreover, many phytochemicals can attenuate virulence factors without exerting strong selection pressure on bacterial survival, further lowering the risk of resistance development. For instance, curcumin and berberine have been shown to inhibit biofilm formation and quorum sensing at sub-inhibitory concentrations, reducing bacterial fitness and pathogenicity without necessarily promoting resistance [158]. That said, slow adaptive mechanisms against phytochemicals have been observed in certain conditions. For example, long-term exposure to sub-lethal concentrations may lead to overexpression of general stress response proteins, efflux transporters, or modifications in membrane composition, which can confer some level of tolerance [36]. However, these responses are typically non-specific and reversible upon removal of the compound, unlike the stable, heritable resistance seen with antibiotics.

## 9. Strategic Selection of Antibacterial Agents for Implant Surface Modification

Among the available strategies, antibiotic, metal nanoparticle, and phytochemical-based coatings each offer distinct advantages and limitations. Comparison of surface coatings is tabulated in Table 5. The selection of appropriate antibacterial coatings for biomaterials plays a pivotal role in ensuring the long-term success of medical implants by preventing infections and supporting tissue integration.

Antibiotic coatings have long been employed due to their potent and targeted antimicrobial activity. Antibiotic coatings, including agents like gentamicin, vancomycin, and ciprofloxacin, are well established for effectively preventing bacterial infections, particularly in post-surgical scenarios. These coatings have demonstrated high clinical efficacy in infection control. However, their clinical application is increasingly constrained by the emergence of antibiotic-resistant bacterial strains, rapid degradation or burst release, and potential cytotoxic effects at high concentrations [158]. Moreover, the repeated use of antibiotics may disrupt local microbiota and elicit immune responses, further complicating healing and integration. Nonetheless, their use is increasingly scrutinized due to the emerging risk of antibiotic resistance, potential cytotoxic effects, and reduced efficacy against biofilm-forming bacteria, which are often more difficult to eradicate [159].

Metal/metaloxide nanoparticle coatings, particularly those incorporating silver, copper, or zinc, provide broad-spectrum antibacterial effects and can promote wound healing and cell proliferation. These coatings are particularly valuable for their effectiveness against multi-drug-resistant bacteria and biofilm formation, while also contributing to osseointegration enhancement. Despite their effectiveness, metal-based coatings pose risks including potential cytotoxicity, inflammatory responses, and metal ion release, which can lead to systemic toxicity [157]. These coatings often suffer from uncontrolled ion release, long-term cytotoxicity, and possible environmental accumulation. The risk of bacterial adaptation to metal ions also raises concerns regarding resistance, albeit via different mechanisms than antibiotics [157]. Furthermore, high production costs and complex synthesis procedures present practical challenges to widespread clinical adoption.

In contrast, phytochemical-based coatings leverage the antimicrobial potential of natural plant-derived compounds, such as flavonoids, alkaloids, tannins, and essential oils. These agents not only exhibit multifaceted antibacterial mechanisms, including membrane disruption and enzyme inhibition, but also offer antioxidant and anti-inflammatory properties that support tissue regeneration [155]. Importantly, phytochemicals often demonstrate lower toxicity, biodegradability, and reduced likelihood of resistance development. Their eco-friendly origin and compatibility with diverse surface modification techniques make them attractive for next-generation, multifunctional coatings [76].

Given these comparative advantages, phytochemical coatings are emerging as a favorable alternative for biomaterial applications, especially in the context of antibiotic resistance and the need for biocompatible, sustainable solutions. Nonetheless, challenges remain, including variability in natural compound composition, stability during sterilization, and the need for controlled, long-term release. Addressing these issues through standardized extraction, nano-formulation, and advanced delivery systems will be key to translating plant-based coatings into clinical use.

## 10. Conclusions

The selection of antimicrobial agents for coating medical implants and biomaterials is critical to preventing infections and reducing the risk of bacterial resistance. Antibiotic coatings remain an effective, targeted intervention in clinical settings, particularly in post-surgical scenarios, yet their utility is increasingly compromised by bacterial resistance, burst release, cytotoxic side effects, and microbiota disruption. Metal/metaloxide nanoparticle coatings, including silver, zinc, and copper, offer powerful, broad-spectrum antimicrobial activity and biofilm inhibition, with added benefits for osseointegration. However, their long-term success is limited by challenges such as ion-mediated cytotoxicity, inflammatory responses, environmental accumulation, and manufacturing complexity. Conversely, phytochemical-based coatings harness bioactive plant-derived compounds to deliver multifunctional advantages—antibacterial, antioxidant, anti-inflammatory, and osteogenic—with improved biocompatibility and sustainability. Yet, obstacles such as compositional variability, sterilization stability, and controlled release remain to be addressed. Given the urgent need to combat antibiotic-resistant and biofilm-associated infections, phytochemical-based coatings show great promise as next-generation biomaterial solutions. Future efforts should focus on standardizing natural extract compositions, integrating nano-formulations, and engineering advanced delivery platforms that ensure sustained release and clinical viability. By leveraging the strengths of each approach, while mitigating their limitations, combinatorial and multifunctional coating strategies could offer the most effective path forward for enhancing implant safety and performance.

## Figures and Tables

**Table 1:** Coating techniques for implants and biomaterials.

Coating Technique	Description	Advantages	Common Applications	Implant Material	Reference
Physical Vapor Deposition (PVD)	A vacuum-based process that deposits thin films onto a substrate by vaporizing a solid material.	- Strong adhesion - Wear and corrosion resistance	Orthopedic and dental implants	Titanium alloys, Stainless steel	[26,27,28]
Chemical Vapor Deposition (CVD)	A chemical process used to deposit high-purity coatings on implant surfaces.	- Dense, uniform coating - Good corrosion resistance	Biomedical sensors, stents, implants	Titanium alloys, Cobalt-Chrome alloys	[29,30,31,32]
Plasma Spray Coating	Uses a high-temperature plasma torch to spray molten coating material onto a surface.	- Thick coatings - Enhances bone bonding	Hydroxyapatite coatings for bone implants	Titanium, Cobalt-Chrome alloys	[33,34,35,36]
Electrophoretic Deposition (EPD)	Applies an electric field to deposit charged particles from a suspension onto a surface.	- Uniform film formation - Adjustable thickness	Bioceramic coatings, drug delivery coatings	Titanium, Stainless steel	[37,38,39]
Dip Coating	Involves immersing an implant into a coating solution, then withdrawing it to form a uniform layer.	- Simple and cost-effective - Scalable for mass production	Drug-loaded coatings, antibacterial coatings	Titanium, Stainless steel	[26,27,28,40]
Spin Coating	Deposits a thin liquid film onto a spinning surface to achieve uniformity.	- Precise thickness control - Smooth, uniform coatings	Bioactive coatings for sensors and micro-devices	Titanium, Polymers	[29,30,31,32]
Sol-Gel Coating	A solution-based technique where a liquid precursor undergoes gelation to form a thin film.	- Low-temperature processing - Can incorporate bioactive molecules	Bone implants, bioactive glasses, antibacterial coatings	Titanium, Bioactive glass	[33,34,35,36]
Hydroxyapatite (HA) Coating	A calcium phosphate-based bioactive ceramic coating to improve osseointegration.	- Biocompatible - Promotes bone growth	Orthopedic and dental implants	Titanium, Titanium alloys	[37,38]

**Table 2:** Antibiotics as antimicrobial compounds for implant coatings.

Antibiotic	Description	Methods of Coating Application	Common Applications	Side Effects	Reference
Gentamicin	Broad-spectrum aminoglycoside effective against Gram-negative bacteria.	Dip-coating, Electrophoretic Deposition (EPD), Plasma Spray Coating	Orthopedic implants, dental implants, catheters	Nephrotoxicity, Ototoxicity, Neuromuscular blockade	[53,54]
Vancomycin	Glycopeptide antibiotic effective against Gram-positive bacteria, including MRSA.	Sol-gel coating, Layer-by-layer assembly, Spray Coating	Bone implants, joint prostheses, vascular grafts	Nephrotoxicity, Red Man Syndrome, Ototoxicity	[55,56]
Ciprofloxacin	Fluoroquinolone with broad-spectrum activity, effective against biofilm-forming bacteria.	Plasma Spray Coating, Dip Coating, Electro spraying	Titanium-based orthopedic implants, dental implants	Tendonitis, Peripheral neuropathy, GI disturbances	[57,58]
Rifampin	Effective against biofilm-associated Staphylococcus species, often combined with other antibiotics.	Layer-by-layer assembly, Sol-gel Coating	Coating on titanium and polymer-based implants	Hepatotoxicity, GI discomfort, Red/orange discoloration of bodily fluids	[59,60]
Doxycycline	Tetracycline-class antibiotic with anti-inflammatory properties.	Dip-coating, Electrophoretic Deposition (EPD)	Orthopedic implants, bone grafts	Photosensitivity, GI upset, Esophagitis	[61,62]
Tobramycin	Aminoglycoside is used against Gram-negative bacteria, particularly Pseudomonas aeruginosa.	Spray Coating, Sol-gel, Electrospinning	Dental and orthopedic implants	Nephrotoxicity, Ototoxicity, Neuromuscular blockade	[63,64]
Clindamycin	Lincosamide antibiotics are effective against anaerobic and Gram-positive bacteria.	Dip-coating, Plasma Spray Coating	Bone implants, titanium implants	Pseudomembranous colitis, GI disturbances, Rash	[57,65]
Linezolid	Oxazolidinone-class antibiotic used against multidrug-resistant Gram-positive bacteria.	Layer-by-layer assembly, Electrophoretic Deposition (EPD)	Joint prostheses, fracture fixation devices	Myelosuppression, Peripheral neuropathy, Serotonin syndrome	[66,67]

**Table 3:** Metal/metaloxide nanoparticles as antimicrobial compounds for implant coatings.

Coatings	Antimicrobial Agent	Research Findings/Results	Advantages	Challenges	Reference
Silver-based (AgNPs) coatings	Silver nanoparticles (AgNPs)	- Significant reduction in bacterial adhesion and growth.	- Broad-spectrum antimicrobial properties.	- Potential cytotoxicity at higher concentrations.	[79,80]
		- Strong antimicrobial activity against Gram-positive and Gram-negative bacteria.	- Enhances osteointegration.	- Limited long-term stability.	
Copper-Based Coatings	Copper oxide (CuO), Copper nanoparticles	- Effective against both bacterial and fungal infections.	- High antimicrobial efficacy.	- Potential toxicity to human cells at high concentrations.	[81,82]
		- Enhanced biofilm prevention.	- Supports tissue healing.		
		- Fast-acting antimicrobial effect.	- Selective antimicrobial activity.	- Expensive production process.	
Polydopamine Coatings	No specific antimicrobial agent (self-polymerization of dopamine)	- Antimicrobial effects through phenolic groups.	- Simple and versatile coating process.	- Limited long-term stability.	[83,84,85]
		- Prevents bacterial adhesion.	- Enhances bioactivity of the surface.	- Efficacy depends on concentration.	
Titanium Dioxide (TiO_2_) Coatings	TiO_2_ (Photocatalytic)	- Strong antimicrobial properties under UV light.	- UV-induced antimicrobial activity.	- Requires UV light for activation.	[86,87,88]
		- Antibacterial effects due to photocatalysis.	- Low cytotoxicity.	- Limited efficacy under physiological conditions.	
Hydroxyapatite (HA) with metal and Antimicrobial Agents	Silver, Copper, Antibiotics (e.g., Rifampicin)	- Enhances osteointegration while providing antimicrobial effects.	- Osteoinductive and antimicrobial.	- Limited effectiveness of antimicrobial release over time.	[89,90]
		- Sustained release of antimicrobial agents.	- Enhanced bone regeneration.		
Ceramic Coatings (e.g., Zirconia, Alumina)	Metal oxides (e.g., TiO_2_, ZnO)	- Antibacterial properties due to metal oxide presence.	- High wear resistance.	- Difficult to fabricate on certain substrates.	[53,91,92,93]
		- High mechanical strength.	- Enhanced stability in harsh environments.	- Limited flexibility in coating design.	

**Table 4:** Plant phytochemicals in biomaterial coatings: advantages and disadvantages.

Phytochemical	Source	Mechanism in Bone Healing	Advantages	Disadvantages	Reference
ssCurcumin	Turmeric (Curcuma longa)	Enhances osteoblast differentiation, anti-inflammatory, antioxidant properties.	- Promotes bone formation	- Poor bioavailability	[110,111]
			- Reduces inflammation	- Rapid metabolism and elimination	
			- Antioxidant effects		
Quercetin	Onion, Apples, Berries	Increases osteoblast activity and inhibits osteoclasts.	- Anti-inflammatory	- Low water solubility	[112,113]
			- Antioxidant	- Poor absorption in the gut	
			- Enhances bone mineralization		
Genistein	Soybeans	Phytoestrogen that mimics estrogen, stimulates osteoblasts.	- Reduces bone loss	- Can interfere with hormone balance	[114,115]
			- Antioxidant and anti-inflammatory	- May not be effective in postmenopausal osteoporosis	
Resveratrol	Grapes, Red wine	Stimulates osteoblast activity, reduces oxidative stress.	- Protects against bone loss	- Poor bioavailability	[116,117]
			- Anti-inflammatory and antioxidant	- Rapid metabolism	
Epigallocatechin Gallate (EGCG)	Green Tea	Enhances osteoblast function, inhibits osteoclast differentiation.	- Antioxidant and anti-inflammatory	- High doses may cause liver toxicity	[33,34]
			- Enhances bone formation	- Poor stability in the body	
Berberine	Berberis species	Enhances osteoblast activity, reduces inflammation.	- Inhibits bone resorption	- Low oral bioavailability	[31,118]
			- Enhances osteogenesis	- Potential gastrointestinal issues	
Catechins	Green Tea	Antioxidant, reduces inflammation, promotes osteoblast activity.	- Supports bone regeneration	- Limited durability in coatings	[119,120]
			- Biodegradable and biocompatible	- Cytotoxicity at high concentrations	

**Table 5:** Comparison of phytochemical, antibiotic, and metal/metaloxide nanoparticle coatings in biomaterials.

Coating Type	Advantages	Disadvantages	Reference
Phytochemical Coatings (e.g., Curcumin, Quercetin, Resveratrol)	- Natural, biocompatible, and biodegradable - Antioxidant and anti-inflammatory properties - Promotes bone regeneration and reduces osteoclast activity - Low risk of bacterial resistance development	- Poor stability and bioavailability - Rapid degradation in physiological conditions - Limited long-term durability in coatings	[76,155]
Antibiotic Coatings (e.g., Gentamicin, Vancomycin, Ciprofloxacin)	- Highly effective against bacterial infections - Prevents post-surgical infections - Established clinical efficacy	- Risk of antibiotic resistance - Potential cytotoxicity - Limited effectiveness against biofilm-forming bacteria	[156]
Metal/metaloxide Nanoparticle Coatings (e.g., Silver, Zinc, Titanium, Copper)	- Strong antimicrobial activity - Long-term stability and durability - Effective against drug-resistant bacteria and biofilms - Enhances osseointegration	- Potential cytotoxicity and inflammatory response - Risk of metal ion release leading to toxicity - Expensive and complex synthesis methods	[157]

## Data Availability

The data that support the findings of this study are available from the corresponding authors upon reasonable request.
